# Immunoregulatory Sertoli Cell Allografts Engineered to Express Human Insulin Survive Humoral-Mediated Rejection

**DOI:** 10.3390/ijms232415894

**Published:** 2022-12-14

**Authors:** Rachel L. Washburn, Taylor Hibler, Gurvinder Kaur, Anna Sabu-Kurian, Alissa Landefeld, Jannette M. Dufour

**Affiliations:** 1 Department of Cell Biology and Biochemistry, School of Medicine, Texas Tech University Health Sciences Center, Lubbock, TX 79430, USA; 2 Immunology and Infectious Disease, Graduate School of Biomedical Sciences, Texas Tech University Health Sciences Center, Lubbock, TX 79430, USA; 3 Department of Medical Education, School of Medicine, Texas Tech University Health Sciences Center, Lubbock, TX 79430, USA; 4 Summer Accelerated Biomedical Research Program, Graduate School of Biomedical Sciences, Texas Tech University Health Sciences Center, Lubbock, TX 79430, USA

**Keywords:** diabetes, insulin, Sertoli cells, complement, genetic engineering, transplantation

## Abstract

An effective treatment and possible cure for type 1 diabetes is transplantation of pancreatic islets. Unfortunately, transplanted islets are rejected by the immune system with humoral-mediated responses being an important part of rejection. Sertoli cells (SC), an immune regulatory cell shown to survive as allografts long-term without immunosuppressants, have the potential to be used as a cell-based gene therapy vehicle to deliver endogenous insulin—a possible alternative to islets. Previously, we transduced a mouse SC line to produce human insulin. After transplantation into diabetic mice, these cells consistently produced low levels of insulin with graft survival of 75% at 50 days post-transplantation. The object of this study was to assess humoral immune regulation by these engineered SC. Both nontransduced and transduced SC survived exposure to human serum with complement in vitro. Analysis of allografts in vivo at 20 and 50 days post-transplantation revealed that despite IgG antibody detection, complement factor deposition was low and grafts survived through 50 days post-transplantation. Furthermore, the transduced SC secreted elevated levels of the complement inhibitor C1q binding protein. Overall, this suggests SC genetically engineered to express insulin maintain their ability to prevent complement-mediated killing. Since inhibiting complement-mediated rejection is important for graft survival, further studies of how SC modifies the immune response could be utilized to advance the use of genetically engineered SC or to prolong islet allograft survival to improve the treatment of diabetes.

## 1. Introduction

Diabetes mellitus, a chronic disease characterized by elevated blood glucose levels, insulin resistance, and/or death of pancreatic islets afflicts over 10% of the world population [1]. Because of this, diabetes patients have elevated blood glucose levels, which cause many comorbidities such as cardiovascular disease, peripheral neuropathy, and lower limb amputation [2,3]. Pancreatic islet transplantation in patients with brittle type 1 diabetes (characterized by large shifts in blood glucose) is performed worldwide and is a safe and effective diabetes treatment [4,5]. As pancreatic islets produce insulin, this procedure replaces damaged islets with functional islets that endogenously produce and secrete insulin in response to real-time glucose levels to achieve normoglycemia. Unfortunately, islet allotransplants can be rejected by the recipient’s immune responses [6,7]. To mitigate transplant rejection and prolong allogeneic islet survival, transplant recipients must take chronic, lifelong immune suppressants [8,9] which have many harsh side effects such as frequent infections, stunted wound healing, cancer development, and even toxicity to the islets themselves [10,11]. Unfortunately, islet grafts are still rejected with this therapy [12].

Interestingly, Sertoli cells (SC), an immunoregulatory testicular cell, have been shown to survive long-term post-transplantation without immune suppressants [13]. The unique immunoregulatory properties of SC make them promising candidates as cellular-based gene therapy vehicles to deliver therapeutic proteins [14,15,16,17,18]. Previously, we have found that mouse and pig SC can be engineered to express functional insulin [17]. Building on previous research, we engineered a mouse SC line (MSC1) to express furin-modified human insulin using a lentiviral vector (MSC1-HI) and found that they produce insulin and survive as allografts in diabetic mice throughout the study [16]. The goal of this study was to examine the role of antibody activation of complement-mediated killing in these MSC1 and MSC1-HI cell grafts.

**Figure 1:**
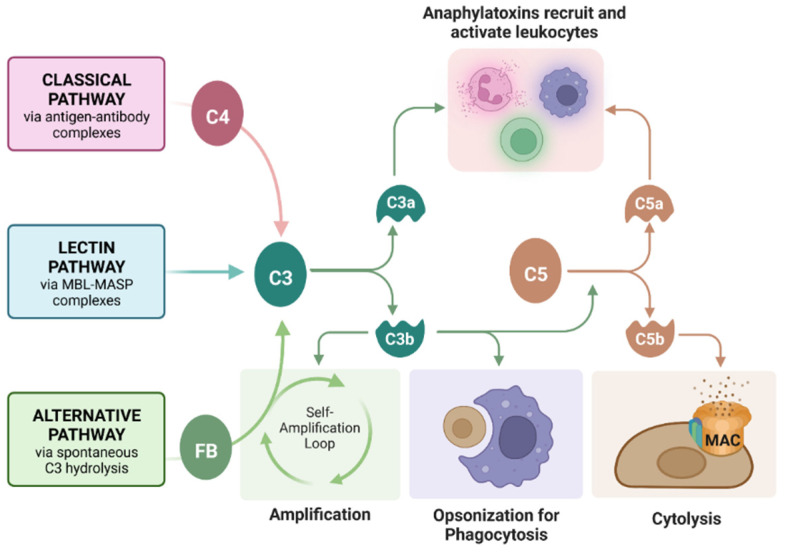
Roles of the complement cascade in graft rejection. Complement can be activated by antibody-bound-antigens recruiting C4 (classical pathway), mannose-binding lectin (MBL)-MBL serine protease (MASP) complexes (lectin pathway, not commonly implemented in graft rejection), or spontaneously by C3 hydrolysis and Factor B (FB) recruitment (alternative pathway). All three pathways converge on the cleavage of C3, which feeds back into the alternative pathway for amplification, opsonizes the grafted cells for phagocytosis, or cleaves C5 to form the membrane attack complex (MAC, C5bC6C7C8C9), causing cell lysis. Anaphylatoxins C3a and C5a recruit and activate leukocytes, inducing inflammation. Figure created with Bio Render.com (accessed on 29 November 2022).

Complement is an important component of transplant rejection. Complement is a cascade of protein cleavages that serves to reject grafts (or kill pathogens in a normal environment) through three primary mechanisms: (1) cytolysis by insertion of a pore complex (membrane attack complex, MAC), (2) phagocytosis of graft cells by phagocytic cells such as macrophages through opsonization with complement fragments C4b and C3b, and (3) inducing chemotaxis of immune cells such as macrophages (phagocytic cells that aid in graft rejection) through release of the anaphylatoxins C3a and C5a (Figure 1) [19]. Previously, we reported that SC survive a human serum (containing antibodies and complement) cytotoxicity assay in vitro and acute complement activation in vivo [20]. However, the effect of complement on engineered SC had not yet been analyzed.

Since the complement system is an important mediator of graft rejection, in this study we tested the hypothesis that these genetically engineered MSC1-HI cells maintain some of the immunoregulatory properties of SC, specifically, the ability to inhibit complement activation, allowing them to survive long-term as allografts in diabetic mice. To determine whether transduced SC survive activated complement, we exposed MSC1-HI cells to human serum containing antibodies and complement in vitro and quantified their survival. We then analyzed MSC1 and MSC1-HI cell grafts collected from diabetic mice at days 20 and 50 post-transplantation for cell survival [16], deposition of antibodies (IgM and IgG), and complement components (C4, C3, FB, and the MAC component C9) to assess their interaction with complement in the long-term. Last, we used ELISA to quantify secretion of complement inhibitors cartilage oligomeric matrix protein (COMP) and C1q binding protein (C1QBP).

## 2. Results

### 2.1. Survival of MSC1-HI Cells Transduced to Express Human Insulin after Exposure to Human Serum Containing Complement

**Figure 2:**
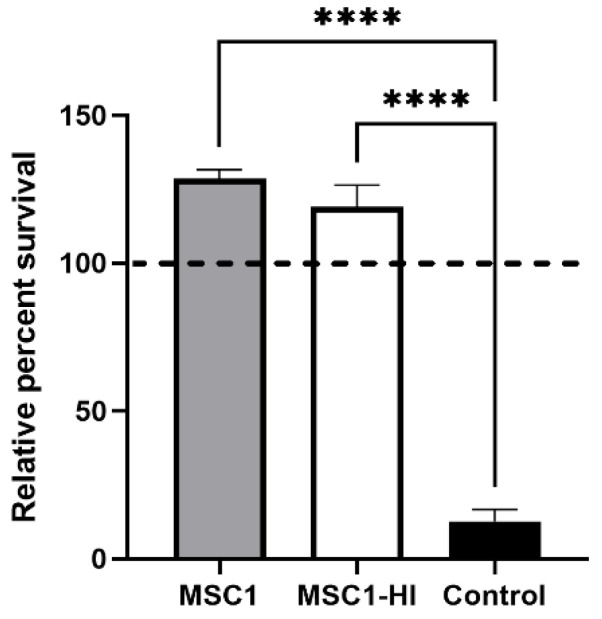
Non-transduced and transduced MSC1 cells survive after exposure to human serum in vitro. MSC1 cells (gray bar), MSC1-HI (white bar), and control cells (PAECs, black bar) were exposed to human AB serum containing antibodies and active complement. Cell survival was assessed by MTT cell viability assay. Cell viability for cells cultured in media only was set to 100% (dashed line) and the relative percent viability was calculated for each cell type. Viability is presented as the mean ± SEM for at least three different experiments. The significance was determined by one-way ANOVA. **** indicates *p* < 0.0001.

To find out whether engineered MSC1 cells maintain the ability to survive after exposure to complement, MSC1 cells transduced with a lentiviral vector to stably express furin-modified human proinsulin (MSC1-HI) [16] were exposed to human AB serum containing antibodies and active complement. This assay was chosen because xenografts face very robust complement-mediated killing, and thus survival of human complement in this assay is indicative of vigorous cell survival. Controls included non-transduced MSC1 cells and porcine aortic endothelial cells (PAEC). PAEC are known to be killed by this assay [21]. MSC1-HI cells exposed to activated human complement survived at 125%, similar to primary SC and MSC1 cells as we reported previously [20], whereas PAEC survival was extremely diminished at 12% (Figure 2). These data imply that transduced SC maintain the complement-survival properties of non-transduced SC in vitro.

### 2.2. Survival of MSC1 and MSC1-HI Cells after Transplantation as Allografts in Mice

**Figure 3:**
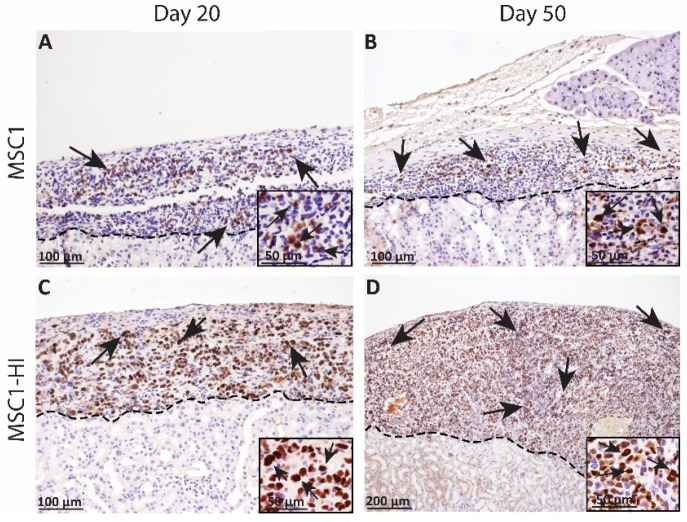
Immunohistochemical analysis of MSC1 and MSC1-HI cell allograft survival. Six million MSC1 (**A**,**B**) or MSC1-HI (**C**,**D**) cells were transplanted under the kidney capsule of BALB/c mice. Grafts were collected on days 20 (**A**,**C**) and 50 (**B**,**D**) post-transplantation and immunostained for the MSC1 cell marker large T-antigen (brown). Insets are a higher magnification and are included for visualization of large T-antigen positive cells. A dotted line separates the graft (above the line) from the kidney (below line). Sections were counterstained with hematoxylin (blue). Arrows indicate examples of positive staining.

In an earlier study, we transplanted six million MSC1 or MSC1-HI cells under the kidney capsule of allogeneic diabetic mice and collected the grafts at days 20 and 50 post-transplantation. Survival was verified with immunohistochemistry for the MSC1 marker large T antigen (Figure 3) [16]. MSC1 grafts survived at 86% (day 20 post-transplantation, Figure 3A) and 70% (day 50 post-transplantation, Figure 3B) while MSC1-HI grafts survived at 88% (day 20 post-transplantation, Figure 3C) and 75% (day 50 post-transplantation, Figure 3D). Although mice did not achieve normoglycemia during the study, cells within the MSC1-HI grafts were positive for insulin in vivo while insulin was not detected in non-transduced MSC1 cells [16].

**Figure 4:**
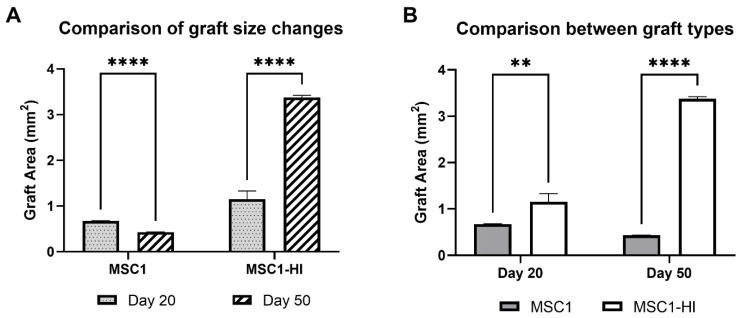
Comparison of graft area between MSC1 and MSC1-HI cell allografts. (**A**) Comparison of changes in graft size were measured for MSC1 and MSC1-HI cells at day 20 (dotted gray bar) and day 50 (striped bar) post-transplantation. (**B**) Comparison of sizes between the graft types at each time point were measured for MSC1 cell (gray bar) or MSC1-HI cell (white bar) grafts (*n* = 3 each) at days 20 and 50 post-transplantation. Asterisks denote comparison between MSC1 and MSC1-HI cells. ** *p* < 0.01; **** *p* < 0.0001. Significance was measured using unpaired *t* test.

Interestingly, the graft area was significantly different between MSC1 and MSC1-HI cells at both timepoints (Figure 4A). Graft area was also significantly different for both MSC1 and MSC1-HI cells between days 20 and 50 post-transplantation (Figure 4B). On day 20 post-transplantation, the average MSC1 graft area was 0.68 mm^2^, which decreased by 64% at day 50 to 0.43 mm^2^ (*p* = 0.0003). The MSC1-HI graft area was 1.16 mm^2^ on average at day 20 and increased by 292% at day 50 to 3.37 mm^2^ (*p* < 0.0001). Comparison of the two graft types at day 20 post-transplantation indicated a log2 fold change of 1.7 (*p* = 0.009) and of 7.8 (*p* < 0.0001) at day 50. Overall, both graft types survived through day 50 post-transplantation with MSC1-HI grafts significantly larger than MSC1 grafts.

### 2.3. Deposition of IgM and IgG on MSC1-HI or MSC1 Cell Grafts

**Figure 5:**
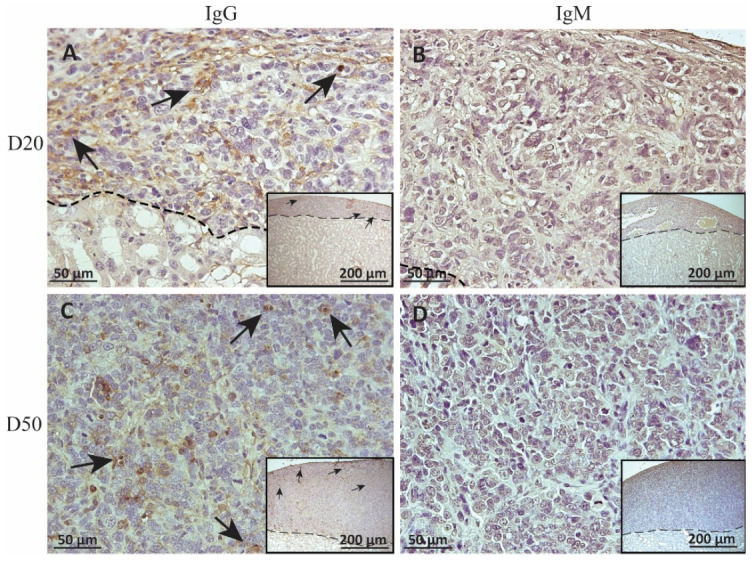
Immunohistochemical analysis of antibody deposition on MSC1-HI chronic timepoint allografts. Graft-bearing MSC1-HI kidneys were collected at days 20 (**A**,**B**) and 50 (**C**,**D**) post-transplantation, and tissue sections were immunostained for IgG (brown; **A**,**C**) and IgM (brown, **B**,**D**). Insets are lower magnification. A dotted line separates the graft from the kidney. Sections were counterstained with hematoxylin (blue). Arrows indicate examples of positive staining.

**Figure 6:**
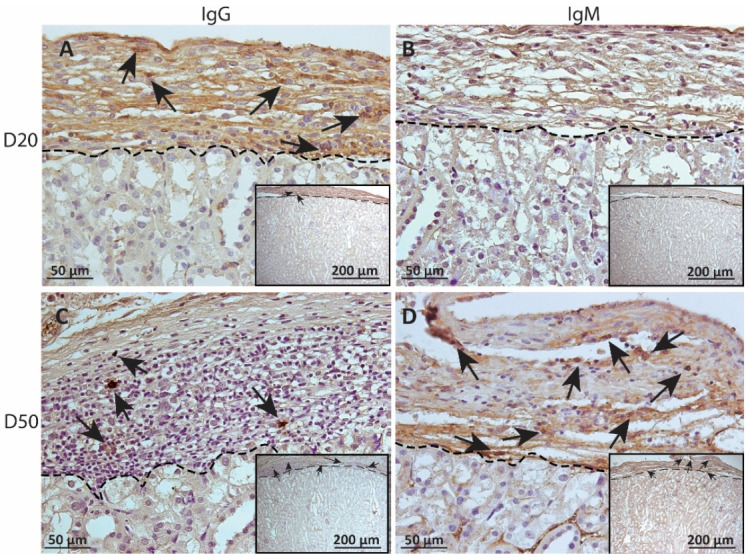
Immunohistochemical analysis of antibody deposition on MSC1 chronic timepoint allografts. Graft-bearing MSC1 kidneys were collected at days 20 (**A**,**B**) and 50 (**C**,**D**) post-transplantation and tissue sections were immunostained for IgG (brown; **A**,**C**) and IgM (brown, **B**,**D**). Insets are lower magnification. A dotted line separates the graft from the kidney. Sections were counterstained with hematoxylin (blue). Arrows indicate examples of positive staining.

**Figure 7:**
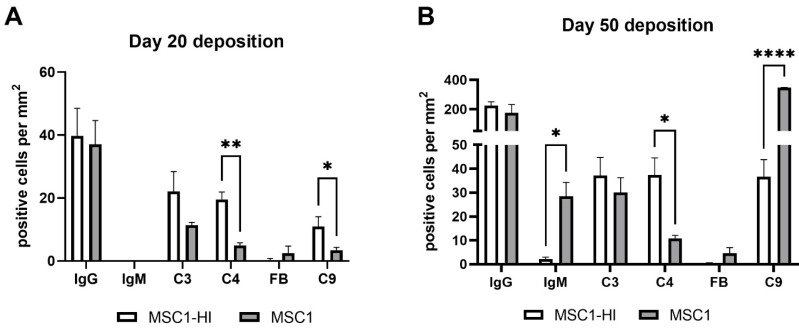
Positively stained cells per mm2 of graft area. MSC1-HI (white bar) and MSC1 (gray bar) were immunostained for IgG, IgM, C3, C4, Factor B (FB) and C9 (pore component of MAC) at day 20 post-transplantation (**A**) and day 50 post-transplantation (**B**). Positively stained cells were counted, and counts were divided by the total graft area to normalize counts between various graft sizes. Asterisks denote significance through unpaired *t*-test with Welch’s correction. * *p* < 0.05. ** *p* < 0.01. **** *p* < 0.0001.

To measure alloantibody binding to transplanted cell surfaces, which can activate complement-mediated rejection, chronic MSC1-HI or MSC1 cell graft tissue was stained for IgG or IgM. In both MSC1-HI and MSC1 grafts, IgG staining was low, at under 40 positive cells per mm^2^ graft area detected at day 20 post-transplantation (Table 1; Figure 5A, Figure 6A and Figure 7A). At day 50 post-transplantation, IgG deposition increased in both graft types with over 150 positive cells per mm^2^ graft area in both graft types (Table 1; Figure 5C, 6C and 7B). No IgM was detected in MSC1-HI or MSC1 cell grafts at day 20 post-transplantation (Table 1; Figure 5B; Figure 6B and Figure 7A) and levels remained nearly zero in MSC1-HI cell grafts (about 2 positive cells per mm^2^ graft area) and was very low in MSC1 cell grafts (under 30 positive cells per mm^2^ graft area) at day 50 post-transplantation (Table 1; Figure 5D, 6D and 7B). Lower IgM was detected in both grafts as compared to IgG (Table 1).

### 2.4. Deposition of Complement Factors C3, C4, FB and C9 in MSC1 or MSC1-HI Cell Grafts

**Figure 8:**
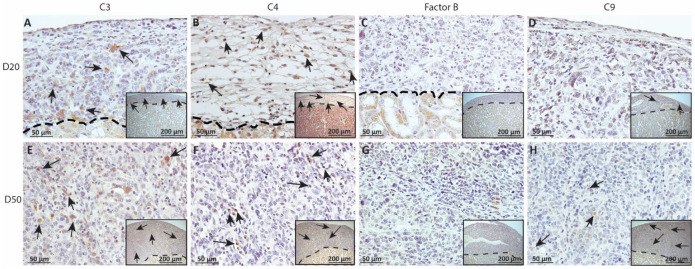
Immunohistochemical analysis of complement fragment deposition on MSC1-HI chronic timepoint allografts. Graft-bearing MSC1-HI kidneys were collected at days 20 (**A**–**D**) and 50 (**E**–**H**) post-transplantation and tissue sections were immunostained for C3 (brown; **A**,**E**), C4 (brown; **B**,**F**), FB (brown; **C**,**G**), or MAC (brown; **D**,**H**). Insets are lower magnification. A dotted line separates the graft from the kidney. Sections were counterstained with hematoxylin (blue). Arrows indicate examples of positive staining.

**Figure 9:**
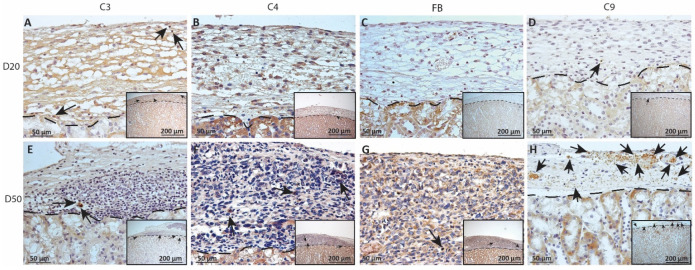
Immunohistochemical analysis of complement fragment deposition on MSC1 chronic timepoint allografts. Graft-bearing MSC1 kidneys were collected at days 20 (**A**–**D**) and 50 (**E**–**H**) post-transplantation and tissue sections were immunostained for C3 (brown; **A**,**E**), C4 (brown; **B**,**F**), FB (brown; **C**,**G**), or MAC (brown; **D**,**H**). Insets are lower magnification. A dotted line separates the graft from the kidney. Sections were counterstained with hematoxylin (blue). Arrows indicate examples of positive staining.

As complement has been implicated in chronic graft rejection, MSC1-HI and MSC1 allografts were immunostained for the iconic complement components C3, C4, FB and C9 (pore component of MAC) at days 20 and 50 post-transplantation (Figure 7, Figure 8 and Figure 9, and Table 2). C3, C4 and C9 staining was initially low on day 20 (Figure 7A) in the MSC1-HI grafts, at around 10 to 25 positive cells; this number increased to about 40 positive cells at day 50 post-transplantation (Figure 7B and Figure 8; Table 2). However, FB staining was low-to-none, with a max of two positive cells noted (Table 2, Figure 8). MSC1 cell grafts showed relatively low C4 and C3 staining consistently, with under 15 positive cells at day 20 post-transplantation and under 35 positive cells at day 50 post-transplantation (Table 2; Figure 7 and Figure 9A,B,E,F). FB was barely detected at values under five positive cells at both timepoints (Table 2; Figure 7 and Figure 9C,G). However, C9 positive cells on MSC1 grafts increased from less than five positive cells to over 200 positive cells (Table 2; Figure 7 and Figure 9D,H), while C9 staining on MSC1-HI grafts was very light.

### 2.5. Protein Expression of COMP and C1QBP by MSC1 and MSC1-HI Cells

**Figure 10:**
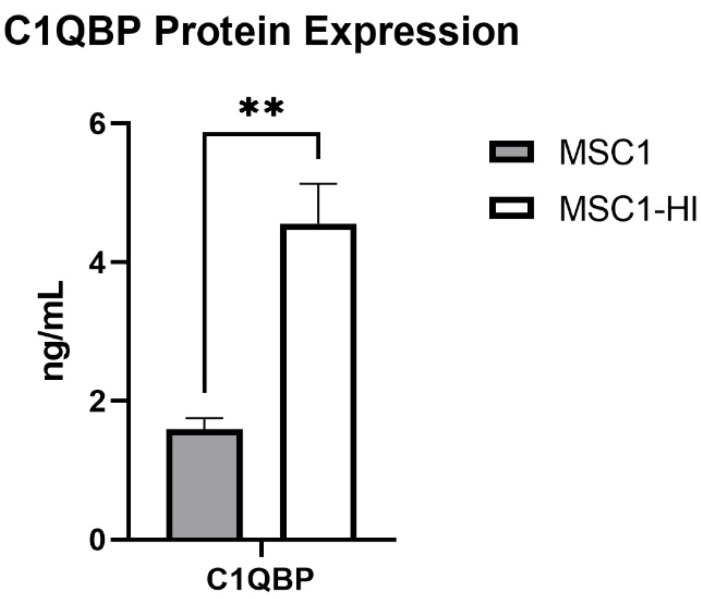
MSC1 and MSC1-HI cells express and secrete protein for complement inhibitor C1QBP. Conditioned media from MSC1 cells (gray bar) or MSC1-HI cells (white 7 bar) were analyzed for protein levels of the complement inhibitor C1QBP, which were expressed at 1.593 ± 0.161 ng/mL by MSC1 cells and at 4.560 ± 0.541 ng/mL by MSC1-HI cells. Asterisks denote significance through unpaired *t-*test with Welch correction. ** *p* < 0.01.

Previously, our group reported that primary mouse SC and MSC1 cells express mRNA for 14 complement inhibitory proteins (CIP) [20]. Of these, MSC1 cells expressed mRNA for C1QBP at significantly elevated levels compared to primary mouse SC (1.205 ± 0.092 ng/mL, *p* = 0.002) [20]. Here, we used ELISA to quantify protein expression of COMP and C1QBP in MSC1 and MSC1-HI conditioned media. COMP secretion was not detected by MSC1-HI or MSC1. MSC1 cells express C1QBP at 1.593 ± 0.161 ng/mL and MSC1-HI cells express C1QBP at significantly higher levels, 4.560 ± 0.541 ng/mL (*p* < 0.01) (Figure 10). Protein levels for both COMP and C1QBP were consistent with the mRNA expression by MSC1 cells [20].

## 3. Discussion

Humoral based complement-mediated rejection still stands as a barrier to long-lived allograft survival. In fact, chronic immune suppression is needed to prevent graft rejection and antibody-mediated response is the most common cause of late graft failure [22]. This is equally true for pancreatic islet transplantation, which could be used to treat, and possibly even cure, type I diabetes [23]. Thus, alternative approaches that reduce the amount of immunosuppressants required and deliver insulin are needed. Given the unique immunoregulatory properties of SC that allow them to survive long-term as allografts and xenografts, we genetically engineered SC to deliver human insulin as a possible treatment for diabetes. Previously, we have found that primary, non-engineered SC survive antibody-mediated complement activation and express several complement regulatory proteins [20]. However, it is not known whether SC engineered to deliver insulin retain these properties. In the current study, we found that the engineered MSC1-HI cells secreted increased levels of C1QBP and were resistant to complement-mediated killing in vitro. Analysis of the MSC1-HI allografts for evidence of the humoral response indicated that, even though IgG antibody and complement components were detected within the grafts, the MSC1-HI cells survived, and the grafts increased in size by day 50 after transplantation. This demonstrates that the insulin expressing SC maintain their ability to prevent complement-mediated killing. Further study on how SC modify the immune response could be utilized to improve graft survival.

The possibility of using SC genetically engineered to deliver insulin or other therapeutic factors as a cell-based gene therapy is attractive since as an immune privileged cell, SC have been shown to survive long-term when transplanted as allografts in mice and rats and as xenografts of pig SC transplanted into rats and dogs without the use of immunosuppressants [24,25,26]. This idea was first explored by Dufour et. al., using mouse SC from GFP transgenic mice where it was found that SC retained the ability to both express GFP and survive as allografts for at least 60 days [15]. Since then, rat, mouse, and pig SC were genetically modified via an adenoviral vector to produce human insulin [17,27]. These insulin-expressing SC were transplanted into diabetic severe combined immunodeficient (SCID) mice, which resulted in reduced blood glucose for up to five days, demonstrating the production of functional insulin by SC; however, since adenoviral vectors do not provide stable integration of the insulin gene, the effect was not permanent [17]. Moreover, these genetically modified SC produced not only insulin, but also C-peptide, a product of insulin processing recently found to be biologically significant [28]. C-peptide has been shown to be preventative against complications of long-term glucose dysregulation such as endothelial dysfunction [29], kidney damage [30], and blindness [31]. Combined, these studies suggest that SC can deliver a therapeutic protein as a potential treatment of diabetes.

Based on the success with the adenoviral vector, we genetically modified the mouse SC line, MSC1 cells, to produce insulin using a lentiviral vector, which allows for stable integration of the proinsulin gene. Initially, MSC1 cells were transduced to express human insulin (MSC1-HI cells). This transduction was validated in vitro by expression of insulin mRNA and protein [16]. When allotransplanted under the kidney capsule of diabetic mice, 75% of MSC1-HI cell grafts survived throughout the duration of the experiment, 50 days post-transplantation. Although insulin in MSC1-HI grafts was detected, mice did not reach normoglycemia. This could be due to either low insulin secretion and/or decreased binding of human insulin to mouse insulin receptors [18,32,33,34]. Still, human insulin was detected through day 50 post-transplantation indicating lower, but sustained, levels of insulin production. Later, MSC1 cells were transduced with a modified lentiviral vector containing the mouse insulin gene and were confirmed to express insulin mRNA and protein [18]. Recipient diabetic SCID and BALB/c mice both had decreased blood glucose levels initially (days 1 to 4) with SCID mice experiencing another decrease in blood glucose levels by day 50 post-transplantations and one diabetic BALB/c mouse undergoing a return to normoglycemia at day 70 post-transplantation [18]. Though adequate insulin production still needs to be optimized and glucose regulated insulin secretion would be ideal, these experiments demonstrate that SC retain their protective properties through genetic modification and might represent a viable option for treating diabetes. However, more information is needed on how engineered SC respond to immune activation.

One of the most notable takeaways from the current study is that genetically modified SC survive complement while producing insulin in vitro and in diabetic mice. To understand how SC are surviving initial complement exposure, within the hyperacute range of transplant rejection, we exposed MSC1 cells, MSC1-HI cells, and control cells (pig aortic endothelial cells, PAEC) to activated human complement in vitro. PAEC are an appropriate control for complement-killing since xenografts (donor and recipient are of different species) are very quickly killed by hyperacute complement-mediated cell lysis [9]. In this experiment, we found that while MSC1-HI cells survived with increased cell viability at 125%, control cell survival was significantly decreased to 12%.

Additionally, we examined their survival against chronically activated complement in vivo by analyzing MSC1 and MSC1-HI cell grafts collected from diabetic BALB/c mice for antibody and complement fragment deposition at days 20 and 50 post-transplantation. Previously, we reported MSC1 and MSC1-HI cell graft survival at day 50 post-transplantation at 75% and 70%, respectively [16]. Here, we found that the MSC1-HI cell grafts were consistently far larger (171% larger at day 20 and 780% at day 50 post-transplantation on average) than the MSC1 cell grafts. This observation may be attributed to the presence of insulin from the MSC1-HI cells, since insulin encourages SC proliferation and growth [35,36]. This phenomenon should be further studied in primary SC to ascertain if insulin production by SC also effects their proliferation after transplantation.

Immunohistochemical analyses of the grafts for antibody binding found IgG deposition at similar levels in MSC1-HI grafts versus MSC1 cell grafts. However, IgM was not detected deposited on MSC1-HI cells while it was detected on MSC1 cells, albeit at low levels. As antibody-antigen complexes can activate the classical pathway of complement, we also performed immunohistochemical analyses for deposition of the complement components C4 (classical pathway activation), FB (alternative pathway activation and amplification loop), C3 (convergent point of all activation pathways) and C9 (pore component of MAC, cytolytic response). C4 deposition at day 50 was significantly higher in MSC1-HI cell grafts as compared to MSC1 cell grafts while C3 was not significantly different. Interestingly, FB deposition was low-to-none on bot MSC1-HI and MSC1 cell grafts. As FB is an integral part of the amplification response, this indicates inhibition of the positive feedback loop of the complement cascade. Regarding the terminal pathway, C9 deposition was low in both sets of grafts at day 20 and lower in MSC1-HI cell grafts at day 50 post-transplantation when compared to MSC1 cell grafts. A decrease in MAC insertion could explain the larger size of MSC1-HI grafts at day 50 post-transplantation. What is remarkable is that most of the cells in these grafts are surviving at 50 days post-transplantation without immune suppressants, even with antibody binding and complement activation, indicating chronic inhibition of the complement cascade. This is in line with our previous study, which detected inhibition of complement-mediated destruction in mouse SC allografts at hyperacute and acute-rejection timepoints [20].

In addition to rejecting islet allografts, dysregulated complement activation also plays a considerable role in diabetes complications and pathogenesis. Complement components such as C3, Factor D, anaphylatoxins, and MAC are associated with sustaining low-grade inflammation, recruiting proinflammatory immune cells, and are linked to diabetic microvascular complications (reviewed in [37]). In fact, increased levels of complement activation over time have been correlated with many of the pathologies of diabetes and insulin resistance, as summarized in Table 3 [37]. Clearly, dysregulated complement is an important, and often understudied, immune component that can wreak havoc in patients with metabolic disorders. Controlling complement activation in patients with chronic diabetes may be a potential avenue in which to treat diabetes and prevent diabetic complications. Therefore, further analysis of SC complement regulation could identify novel mechanisms of complement inhibition beneficial to treat diabetes complications.

Previously, we identified RNA for seven CIP expressed by MSC1 cells (as compared to 14 CIP expressed by primary mouse SC) by microarray analyses [20]. These inhibitors are C1 inhibitor (C1INH), C1qBP, pentraxin (PTX3), sushi domain protein 4 (SUSD4), Crry, CD46, CD59 and clusterin. Expression of most CIP was significantly elevated in primary mouse SC as compared to MSC1 except for C1QBP and CD59 [20]. We previously confirmed mRNA expression of CD59 by MSC1 cells with qRT-PCR [48], so in this study we used ELISA to confirm and quantify protein expression of C1QBP. MSC1 cells express 1.593 ± 0.161 ng/mL of C1QBP, which is comparable to that expressed by primary mouse SC (1.205 ± 0.092 ng/mL) [20]. Of particular interest is that MSC1-HI cells were found to express significantly elevated levels of C1QBP (4.560 ± 0.541 ng/mL) as compared to MSC1 cells. C1QBP is an inhibitor of the classical pathway of complement activation. C1q starts the initiation of complement when it recognizes graft-bound antibodies (IgG or IgM) [49]. Furthermore, C1q has roles outside of direct complement inhibition including regulation of immune cells including effector T cell proliferation and activation [50]. C1QBP expression is also strongly associated with tumor cell proliferation, chemotaxis, and cancer metastasis [51,52,53]. Thus, the elevated expression of C1QBP could either be to increase protection against complement, to suppress effector immune cell responses, to allow for MSC1-HI cell growth, or even a mix of each of these functions. As the MSC1-HI grafts did increase in size, it is possible that C1QBP may be aiding in that process.

Future studies should quantify and compare complement proteins contained in the graft and in serum of the recipient animal to assess the role of local complement. Additionally, future studies should ascertain how to achieve therapeutic and glucose regulated expression levels of human insulin by primary SC in vivo to provide normoglycemia long-term in diabetic mice while preventing graft rejection. Utilizing SC in cell-based delivery of endogenous insulin could overcome the major hurdles of transplant rejection and toxic immune suppression to provide more effective treatment of diabetes mellitus.

## 4. Materials and Methods

### 4.1. Animals

All animals were housed and maintained at appropriate conditions in adherence to the approved Institute for Laboratory Animal Research Care, Use of Laboratory Animals, Texas Tech University Health Sciences Center Institutional Animal Care and Use Committee guidelines and protocols of the National Institutes of Health (IACUC protocol 05019).

### 4.2. SC Transduction

The MSC1 cell line was derived from a SC tumor in transgenic C57BL/6 × SJL mixed hybrid mice carrying a transgene containing DNA encoding both small and large T antigen of the SV40 virus fused to the promoter for human Mullerian inhibiting substance [54]. The Mullerian inhibiting substance promoter was used to target the transgene to SC in males. MSC1 cells were transduced using a recombinant lentiviral vector that carried furin-modified human proinsulin cDNA and ZsGFP under the control of the elongation factor 1alpha promoter (EhI-Zs), as previously described in Kaur et al., 2014 [16]. Transduced cells, MSC-EhI-Zs (MSC1-HI), were cultured in DMEM with 5% fetal bovine serum (FBS) and G418 (1000 μg/mL) [16]. Non-transduced MSC1 cells (MSC1) were used as controls and were cultured in DMEM with 5% FBS only. Transduction was validated using RT-PCR for human insulin, immunocytochemistry for insulin and GFP, and a human insulin ELISA as previously described [16].

### 4.3. Cell Preparation and Transplantation

Prior to transplantation, MSC1 and MSC1-HI cells were cultured and aggregated in non-tissue culture plates using Ham’s F10 media containing supplements for 48 h at 37 °C [17]. Six million MSC1 or MSC1-HI cells (estimated using a DNA assay [54]) were transplanted under the left renal subcapsular space of male BALB/c mice rendered diabetic by streptozotocin (270–275 mg/kg of body weight, Sigma Chemical Co., Burlington, MA, USA) injected intraperitoneally 7 days before transplantation, as previously described in Kaur et al., 2014 [16].

### 4.4. Human Serum Complement Cytotoxicity Assay

The in vitro human serum complement cytotoxicity assays were performed similarly to that described previously [21]. Briefly, 2 × 10^5^ MSC1 (*n* = 3), and MSC1-HI (*n* = 3) cells were plated per well on a 24-well tissue culture plates (Sigma Chemical Co.) and cultured overnight in 1 mL supplemented DMEM media + 10% FBS. Then, 0.5 mL of media was removed per well and the cells were incubated at 37 °C for 1.5 h in one of three groups: negative control group (incubated in 0.5mL of serum-free media), complement group (incubated in 0.5mL of pooled AB human serum containing complement, Innovative Research, Inc, Novi, MI, USA), and positive control group (incubated in 0.5mL of 1% Triton X-100 detergent). Following incubation, all media was removed. Survival of cells was measured using the Cell Proliferation Kit MTT (3-[4, 5-deimethylthiazol-2-yl]-2, 5-deiphenyltetrazolium bromide) assay (Millipore-Sigma, Darmstadt, Germany) as previously described [21].

### 4.5. Immunohistochemical Graft Characterization

Allograft (MSC1 and MSC1-HI) bearing kidneys were collected at 20- and 50 days post-transplantation (*n* ≥ 3). Tissue was fixed in Z-fix, embedded in paraffin, and antigen retrieval of tissue sections was performed by microwaving the slides in 1X sodium citrate buffer. Endogenous peroxidase activity was quenched with 3% H_2_O_2_. Immunohistochemistry for large T antigen, IgG, IgM, and complement factors (C4, C3, FB, and MAC) was performed as follows. For cell survival, tissues were incubated with monoclonal mouse anti-SV40 large T antigen antibody (1:100 dilution: BD Biosciences, San Jose, CA, USA). To detect antibody deposition within the grafts, allografts were incubated with goat anti-mouse IgG (1:450 dilution, Bethyl, Montogomery, TX, USA) and goat anti-mouse IgM (1:250 dilution, Bethyl). To detect complement activation, tissue sections were immunostained for complement factors using the following rabbit-anti-mouse antibodies: rabbit polyclonal anti-C4/C4a (1:2000 dilution, Abclonal, Woburn, MA, USA), rabbit polyclonal anti-FB (1:2000 dilution, Abclonal), rabbit polyclonal anti-C3/C3a (1:2000 dilution, Abclonal), and rabbit polyclonal anti-C9 (1:2000 dilution, Abclonal). Sections incubated with large T antigen, IgG or IgM, and complement factor antibodies were incubated with appropriate biotinylated secondary antibodies, either goat anti-mouse (1:200 dilution, Vector Laboratories, Burlingame, CA, USA), horse anti-goat (1:200 dilution, Vector Laboratories), or goat anti-rabbit (1:200 dilution, Vector Laboratories), respectively. All sections were treated with ABC-enzyme complex (Vector Laboratories) and Diaminobenzidine (DAB, Biogenex, Fremont, CA, USA) as chromogen followed by a hematoxylin nuclear counterstain. Positive controls consisted of mouse spleen sections (antibodies) or mouse liver (complement factors). Negative controls included tissue sections that were put through the same procedure without primary antibody.

Graft area was calculated using ImageJ software (US National Institutes of Health, Bethesda, MD, USA, https://imagej.nih.gov/ij/download.html (accessed on 15 November 2022)). Briefly, graft cross sections were imaged at 10× and individual images were stitched together using the Photomerge tool in Photoshop (Adobe, San Jose, CA, USA, https://www.adobe.com/products/photoshop.html (accessed on15 November 2022)) to obtain a full image of the graft. Graft images contained a size bar set at time of image capture with AxioVision software (Carl Zeiss Microimaging, Thornwood, NY, USA, https://www.micro-shop.zeiss.com/en/us/system/software+axiovision-axiovision+program-axiovision+software/10221/ (accessed on15 November 2022)). This scale bar was used to set the scale bar on ImageJ and the area of the graft was calculated. Three cross sections from different areas of each graft were imaged and averaged.

### 4.6. ELISA Quantification of COMP and C1QBP Protein Secretion

Fifteen million MSC1 cells (*n* = 3) or MSC1-LV cells (*n* = 3) were cultured overnight on 150 mm tissue culture plates (Corning Inc, Corning, NY) in 35 mL of DMEM + 10% FBS. Conditioned medium was collected per cell type and assayed for COMP and C1QBP by ELISA (Aviva Systems Biology, San Diego, CA, USA) per the manufacturer’s protocol. In summary, standards or samples (1:1 dilution) were added to the provided precoated wells and incubated at 37 °C for 2 h. Next, the provided biotinylated detector antibody was added per well and incubated for 1 h. Then, wells were washed with the provided wash buffer and avidin-horse radish peroxidase was added per well and incubated for 1 h. Last, the provided TMB (3,3′,5,5′-tetramethylbenzidine) substrate was added, and wells were incubated for 20 min after which stop solution was applied. Plates were read within 5 min at an OD absorbance of 450 nm.

### 4.7. Statistical Analysis

All values are expressed as means ± standard error of mean and were compared using unpaired Welch’s t-test per row with individual variances computed for each comparison and two-stage linear step-up program of Benjamin, Krieger, and Yekutieli. Statistical significance between groups was set at a *p* level of *p* < 0.05. All statistical analyses were performed using Prism9 software (GraphPad by Dotmatics, San Diego, CA, USA).

## 5. Conclusions

Overall, these data demonstrate that transduced SC survive activated human complement in vitro and allotransplant rejection in vivo without immune suppressants in diabetic mice. Taken together, this indicates that SC engineered to express human insulin preserve the ability to modify the immune response and therefore have the potential as an alternative to traditional pancreatic islet transplantation. If further studies are able to provide glucose regulated insulin secretion by engineered SC, this could lead to treatment of diabetes with a decreased requirement for toxic immunosuppressants.

## Figures and Tables

**Table 1:** Deposition of IgG and IgM on MSC1 and MSC1-HI grafts.

Antibody	Day 20 Post-Transplantation		Day 50 Post-Transplantation	
	MSC1-HI	MSC1	MSC1-HI	MSC1
IgG	39.8	37.1	>200	173.3
IgM	0	0	2.2	28.5

Counts are reported as number of positive cells per mm^2^ graft area. Three independent experiments were performed with an *n* = 3 for each time point. The number of IgG and IgM positive cells was determined by blinded counting of the positive cells per section for each time point (*n* = 3). Cell counts were divided by the total graft area to determine the number of positive cells per mm^2^ graft area.

**Table 2:** Complement deposition (C3, C4, FB and C9) on MSC1-HI and MSC1 grafts.

Complement Factor	Day 20 Post-Transplantation		Day 50 Post-Transplantation	
	MSC1-HI	MSC1	MSC1-HI	MSC1
C3	22.1	11.4	37.1	30.1
C4	19.6	4.938	37.4	10.8
FB	0.2983	2.478	0.2	4.625
C9	10.94	3.462	6.67	>200

Counts are reported as number of positive cells per mm^2^ graft area. Three independent experiments were performed with an *n* = 3 for each time point. The number of C4, FB, C3 and C9 (MAC pore component) positive cells was determined by blinded counting of the positive cells per section for each time point (*n* = 3). Cell counts were divided by the total graft area to determine the number of positive cells per mm^2^ graft area.

**Table 3:** Complement components in diabetes mellitus.

Complement Component	Levels in Diabetes	Effects in Diabetes	Ref.
C1q (CP, activation)	Elevated	Apoptosis of adipocytes, increases infiltration of inflammatory macrophages	[37]
C3 (AP, amplification)	Elevated	Insulin resistance; increases diabetic neuropathy, nephropathy, retinopathy, microangiopathy, and atherosclerosis; causes chronic fibrinolysis and thrombosis; important in beta cell protective autophagy	[38,39]
C3a (anaphylatoxin)	Elevated	Increases inflammation and macrophage recruitment, elevates secretion of insulin by beta cells	[40]
C3aR (receptor)	Elevated	Enhances expression of inflammatory mediators and activation of immune cells	[41]
C3a desArg (anaphylatoxin)	Elevated	Stimulates uptake of glucose, lipid storage, and triglyceride synthesis in adipose tissue; promotes microvascular complications in metabolic disorders	[42,43]
Factor D (AP, amplification)	Elevated	Elevated in obesity, leads to increased activation and amplification through the alternative pathway	[37]
MAC (cytolysis)	Elevated	Promotes endothelial dysfunction and production of ROS; stimulates diabetic complications	[44,45,46]
MASP-2 (LP, activation)	Elevated	Promotes endothelial dysfunction, ROS production, and diabetic vascular complications	[44,45]
CD55 (CIP of C3 and C5)	Reduced	Decreases inhibition of C3 and C5 convertases involved in amplification	[47]
CD59 (CIP of MAC)	Reduced	Decreases inhibition of MAC; maintains stability of lipid rafts, basal insulin secretion, and recycling exocytotic core proteins for insulin release	[44,45,47]

AP: alternative pathway; C3a desArg: inactive form of C3a; C3aR: C3a receptor; CIP: complement inhibitory protein; CP: classical pathway; LP: lectin pathway; MAC: membrane attack complex (C5-9); MASP: mannose-binding protein serine protease; ROS: reactive oxygen species.

## Data Availability

Not applicable.
